# Effect of tooth-bleaching on the carbonate concentration in dental enamel by Raman spectroscopy

**DOI:** 10.4317/jced.53145

**Published:** 2017-01-01

**Authors:** Tatiana Vargas-Koudriavtsev, Óscar-Andrey Herrera-Sancho

**Affiliations:** 1 Dental Faculty, University of Costa Rica. Postgraduate Program in Prosthodontics, University of Costa Rica; 2 School of Physics, University of Costa Rica. Materials Research Science and Engineering Center, University of Costa Rica. Institute for Quantum Optics and Quantum Information, Austrian Academy of Sciences, Innsbruck, Austria

## Abstract

**Background:**

There are not many studies evaluating the effects of surface treatments at the molecular level. The aim of this *in vitro* study was to analyze the concentration of carbonate molecules in dental enamel by Raman spectroscopy after the application of in-office and home whitening agents.

**Material and Methods:**

Sixty human teeth were randomly divided into six groups and exposed to three different home bleaching gels (Day White) and three in-office whitening agents (Zoom! Whitespeed and PolaOffice) according to the manufacturer´s instructions. The concentration of carbonate molecules in enamel was measured prior to and during the treatment by means of Raman spectroscopy. Statistical analysis included repeated measures analysis of variance (*p*≤0.05) and Bonferroni pairwise comparisons.

**Results:**

At home bleaching agents depicted a decrease in the carbonate molecule. This decrease was statistically significant for the bleaching gel with the highest hydrogen peroxide concentration (*p*≤0,05). In-office whitening agents caused an increase in carbonate, which was significant for all three groups (*p*≤0,05).

**Conclusions:**

In-office bleaching gels seem to cause a gain in carbonate of the enamel structure, whilst at-home whitening gels caused a loss in carbonate.

** Key words:**Bleaching, whitening, hydrogen peroxide, carbamide peroxide, Raman spectroscopy, carbonate.

## Introduction

Currently different surface treatments are used in dentistry to modify and improve the optical properties and microstructure of teeth, such as tooth whitening (bleaching), remineralizing treatments and the application of some acids to improve adhesion. There is ample evidence in the literature that describes the effects of these treatments on the surface hardness, structural changes, coloration and adhesion to restorative materials (1-6). However, there are not many studies evaluating these effects at the molecular level which would allow a deeper understanding and a more complete description of the clinical behavior of these materials.

Since Raman spectroscopy is a nondestructive method used to analyze the molecular composition of different substances, it is ideal for analyzing inorganic surface tissue (for example the concentration of molecules of phosphate and carbonate). It is possible to obtain information about minerals through the observation of their energy via excitation of the vibrational modes (7-9). As it is well known, it is possible to excite molecules (for example using electromagnetic radiation from a laser), which absorb and emit some of this energy which is easily measured with commercial photodetectors. A simple way to understand Raman spectroscopy is to observe the types of dispersion occurring when the electromagnetic radiation interacts with different molecules (7,9,10). Sometimes, the energy carried by the incident electromagnetic radiation and the emitted photon energy is equal before and after the collision with the molecule, this case is called elastic scattering or Rayleigh. Other times there is a variation between the initial and final photon energy after the interaction with the molecule. This is called inelastic or Raman scattering. At the atomic level, the probability of Raman scattering to occur is approximately 100 times smaller than for Rayleigh scattering. Raman scattering or emission of radiation energy of these molecules is carried out in the region of the electromagnetic spectrum called near infrared. Also, surface molecules present in dental structures can be characterized from its emission spectrum in the near infrared region known as vibrational spectra. Raman spectroscopy has advantages such as 1) easy preparation of the sample, 2) simple band and spectral analysis, 3) linear response to minerals and chemical concentrations, 4) analysis of samples without any damage, 5) high sensitivity to small structural changes and 6) possibility to choose the amount of the sample to be analyzed. Also, being a non-destructive method (because the specimen must not be altered each time for analysis), the samples can be analyzed before and after treatment, meaning that each specimen can function as its own control (11).

Due to these characteristics, Raman spectroscopy is an optimal technique to investigate dental structure (11-13) and more specifically the effect of dental whitening agents that use hydrogen peroxide or carbamide peroxide (11,14). Both, home and office tooth whitening treatments are promoted as a conservative treatment with few side effects. However, few studies have analyzed the changes in enamel at the molecular level and those that did have very conflicting results. Some researchers suggest that the concentration of the phosphate molecule present in enamel, decreases after the application of bleaching (6,11,12,14), other studies suggest that the molecular concentration remains unchanged (15,16). However, as mentioned before, there is little literature that discusses the topic and research shows irregularities in the spectroscopic method used (9,17). One of such inconsistencies is using silicon as a reference because it is far in the range of the excitation energy of the vibrational modes compared to the molecules present in enamel. In addition, studies estimate the concentration of the molecules in the tooth as the maximum height of the curve observed in the spectrum (peak intensity) in Raman spectroscopy. Whereas the correct way is to estimate the concentration using the area under the curve measured with the spectrometer.

Constantly, new commercial whitening products are advertised having different concentrations of the active agent. These promise to reduce tooth sensitivity and accelerate the whitening process. This is why the molecular analysis of tooth enamel after these treatments is fertile ground for research in dentistry. Several investigations have analyzed the impact of hydrogen peroxide and carbamide peroxide on phosphate present in enamel (9,11,12,14,18), however very few of them describe the effect on carbonate. The aim of our research is to analyze with Raman spectroscopy changes in the concentration of the carbonate molecules after bleaching with different home and in-office bleaching agents, and the effect caused by the use of a remineralizing agent.

## Material and Methods

The present research protocol was approved by the Research Commission of the Dental Faculty at the University of Costa Rica. Sixty sound human teeth, freshly extracted for periodontal and/or orthodontic reasons, were selected. Teeth were cleaned from calculus, debris and blood, inspected for caries and enamel defects and stored in distilled water at 32 °C until required. Specimens were randomly divided into six experimental groups, according to the type of bleaching agent ( Table 1). Within each experimental group, specimens were numbered 1-10, for later statistical purposes.

**Table 1:**
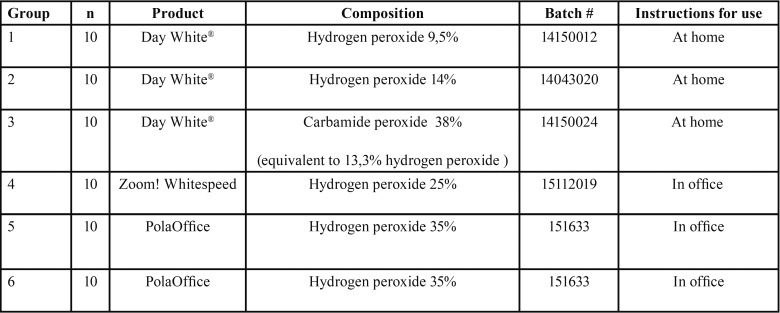
List of treatment groups and materials used.

The most prominent point on the buccal (front) surface of the tooth was set as the working area. It was marked with two lines with a fine diamond bur in order to isolate the area for the application of the bleaching agent and for the readings with the laser focus beam repetitively on the same spot.

Specimens were dried with tissue paper before each whitening procedure taking care not to dehydrate the tooth. For groups 1, 2 and 3, bleaching was carried out twice a day for 30 minutes (at 07:00 hours and 16:00 hours), according to the manufacturer`s instructions. This was done consecutively for 28 days. After each bleaching agent application, specimens were washed and immersed in distilled water in at 32 °C until the next application. Each experimental group was stored separately in multi-compartment boxes. Bleaching was performed by a blind researcher, who wasn´t aware of the treatment conditions of each specimen. After four weeks of continuous bleaching a remineralizing agent was applied for seven days, at the same frequency as the whitener.

Groups 4-6 were bleached with office whitening gels. Group 4 received the whitening agent Zoom! Whitespeed (Philips-USA) and was photo-activated for 15 minutes with a LED light source (420-480nm wavelength), afterwards specimens were cleaned with distilled water and the procedure was repeated one more time with a total of two applications in the first session. One week after the first bleaching session a third application was done under the same experimental conditions.

Group 5 was bleached with PolaOffice (SDI North America Inc.) under the same conditions as group 4, being the only difference the photo-activation time, since the manufacturer recommends only 8 minutes of activation. Group 6 received the same whitening agent as group 5, but it didn`t receive any photo-activation in any of the three applications.

Raman spectra were obtained with a confocal Raman spectrometer (Model Alpha300 R from the company WiTec, Ulm, Germany), fitted with a 100 mW He/Ne laser (785 nm wavelength) and equipped with an Olympus microscope with adjustable confocal hole. Laser diameter was circa 50 μm. These values were verified periodically to ensure that there were no variations due to equipment or optical fiber fluctuations. The reflected light (after interaction with the tooth surface) is collected into a multi-mode fiber, which directs the beam to a spectrometer equipped with a CCD camera. The spectral energy range extends from 0 cm-1 to 3800 cm-1.

Prior to application of bleaching gel, baseline spectra were obtained for all specimens. This way each specimen served as control for itself. Raman spectra were also obtained at the second and fourth week of continuous bleaching for groups 1-3, and after the first and second session of bleaching for groups 4-6.

**Figure 1:**
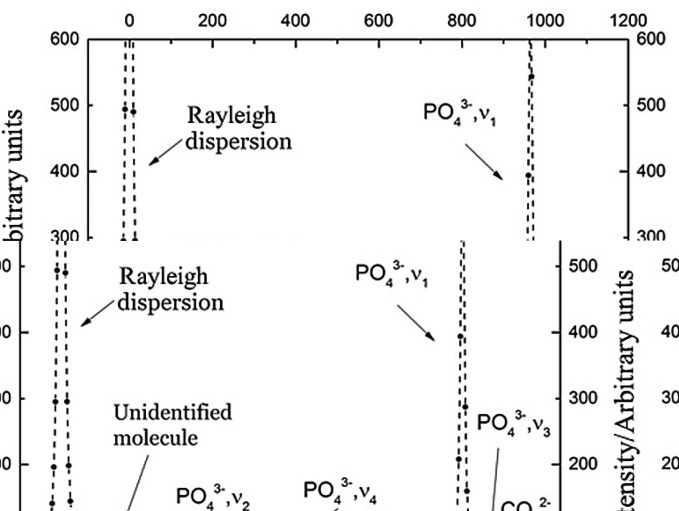
Typical Raman spectrum observed in our experiment.

A typical spectrum would show a peak at 0 cm-1, which corresponds to the energy dispersed in an elastic way (i.e. Rayleigh). Also, one peak can be observed at 1070 cm-1, which is correspondent with carbonate (Fig. 1).

The Raman spectra were taken at one point between the markers on each specimen. One hundred acquisitions were made at each point, with an integration time of 0,5 s.

Immediately after a Raman spectra acquisition, another spectrum was performed on each specimen, switching the laser off, with the same amount of acquisitions and integration time. This spectrum was mathematically subtracted from the one made with the laser in order to obtain a “cleaner” spectrum without the noise of the laser itself.

Each spectrum was imported to the software PeakFit® (Systat Software Inc.) which allows plotting and also a more precise reading of the Wavenumber (horizontal axis) and intensity (vertical axis) values for each spectral peak with its possible adjustments. Also, the base line, type of peak (Gaussian vs Lorentzian), and single points can be considered. The area under the curve of each peak was calculated, since it objectively reflects the intensity of the analyzed molecule.

The data obtained for each specimen for the carbonate molecules (Wavenumber, intensity, area under the curve and Raman spectrum plot), was documented immediately after the spectra was obtained.

-Statistical analysis

Previous to the statistical analysis a normalization with the Rayleigh peaks was performed in order to neutralize the equipment variability towards time and possible laser intensity fluctuations. Descriptive statistics as well as repeated measures analysis of variance (ANOVA) for the carbonate molecules was carried out by of SPSS® v19 (IBM Software). The analysis was performed in order to detect any statistically significant difference among the six experimental groups and the different bleaching weeks. Prior to the multivariate tests and Bonferroni comparisons, a Mauchly sphericity test was carried out in order to confirm that the collected data met the necessary criteria for an analysis of variance.

## Results

**Figure 2:**
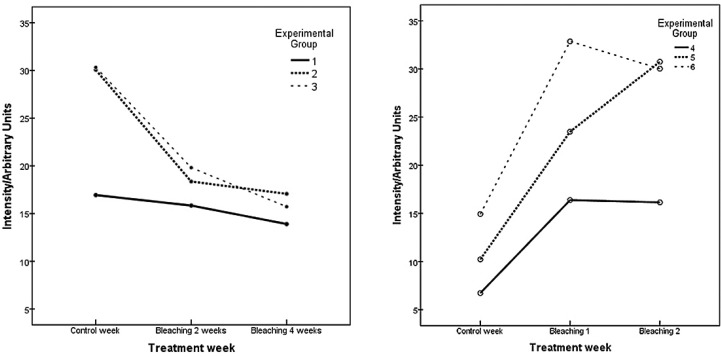
Variation of the carbonate during the treatment weeks.

Figure 2 depicts the behavior of carbonate along the treatment weeks according to each experimental group.

It can be observed that the first three treatment groups showed a progressive decrease of the carbonate molecule along the bleaching treatment. The decrease was stronger in groups 2 and 3. The groups treated with in-office bleaching gels depicted an increase in the molecule. Increase was stronger in the specimens treated with PolaOffice.

 Table 2 shows the multivariate tests for carbonate molecules. It can be seen that molecules concentrations depend on the interaction between the variables “treatment week” and “experimental group”. Carbonate molecules changed their concentration depending on the treatment week, as well as on the type of treatment.

**Table 2:**
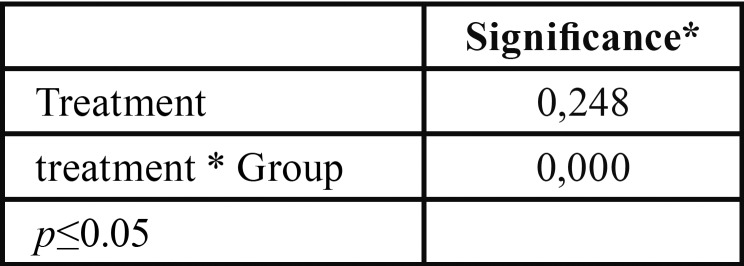
Multivariate test for type of group and treatment week.

According to  Table 3, among the home bleaching agents only group 2 presented a statistically significant reduction in carbonate. All three in-office whitening gels had a statistically significant increase in the studied molecule.

**Table 3:**
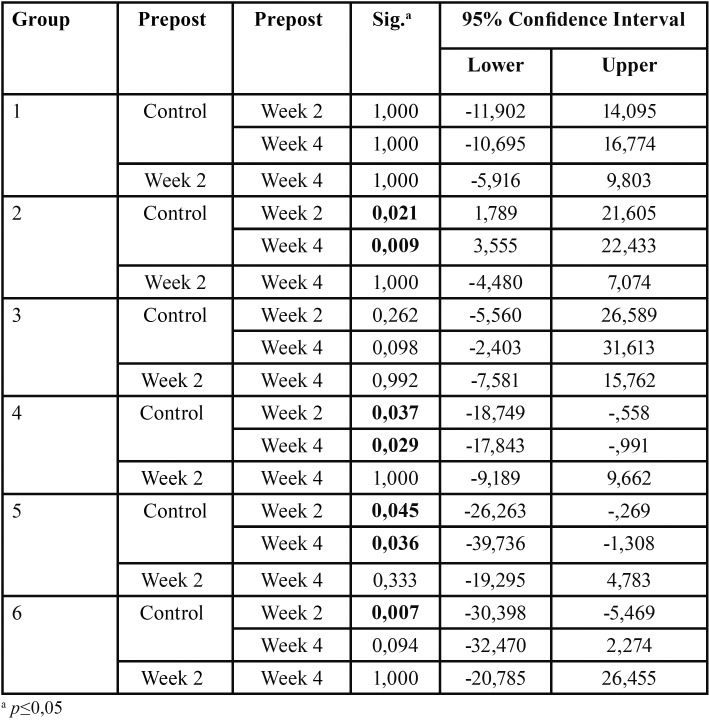
Bonferroni pairwise comparisons.

## Discussion

The main component of dental enamel crystallites is hydroxylapatite (OHAp), which constitutes 98% of its structure (19). There are also different trace materials in enamel, such as chloride, sodium, fluoride, citrate, titanium and carbonate, among others (19-22).

Regarding carbonate, one study revealed that there are vacancies between the crystallites and that enamel molecules are prone to substitutional defects. For example, carbonate ions (CO32-) are able to replace hydroxyl or phosphate ions (22). Also, another study summarizes that carbonate, as a trace element in enamel, decreases crystallographic parameters such as the lattice along the a-axis, and crystallinity and crystal domain size along the c-axis (23,24).

Most of the investigations in bleaching using Raman spectroscopy report the effects on the phosphate molecule. It was of our interest to observe whether carbonate is also affected by the whitening procedure.

The results of our study suggest that bleaching gels that must be applied daily for several weeks, cause a loss of carbonate, in contrast to in-office bleaching agents that are applied in fewer sessions and that increased the content of the studied molecule. Already in the 70`s some authors have related the loss of carbonate with incipient lesions in enamel (21), which is why it might be suggested that superficial treatments that tend to affect less the molecular composition are to be preferred to other options. Jiang *et al.* (11) suggested that even though the demineralization of some bleaching agents is minimal, oxidation still occurs in the organic matter, which affects the integrity of enamel.

Another research group (16) investigated the effect of hydrogen peroxide of diverse pH values on the carbonate concentration in enamel. The authors concluded that the molecule concentration remained unchanged. However, the authors didn´t perform the whitening procedure in a clinically relevant manner, since they only immersed the specimens twice for 30 minutes. Our study carried out the procedure according to the manufacturers´ instructions.

Also, other researchers (25) demonstrated that carbonate decreases its concentration with acidic hydrogen peroxide. This event was observed with Raman spectroscopy as well as attenuated total reflection-Fourier transform infrared spectroscopy (ATR-FTIR). With neutral hydrogen peroxide there were no changes regarding molecular composition, however, acidic whitening agents caused a decrease in the molecules.

Another study (26) proved by means of Raman spectroscopy and attenuated total reflectance-infrared (ATR-IR) spectroscopy that carbonate and phosphate concentration don´t change after employment of home-applied bleaching agents. A possible explanation for these results is that they kept the specimens in natural saliva (of different individuals), which simulated in a better way the real oral environment and also minimized demineralization, but also induced a variable related to the diverse composition of the saliva.

Gotz *et al.* (17) didn`t notice a variation in carbonate after using bleaching strips containing hydrogen peroxide gel at 13% and 16% concentration. Their results were coincident with another investigation (16).

It is worth mentioning that an important advantage of using Raman spectroscopy or X-ray diffraction over attenuated total reflectance-infrared (ATR-IR) spectroscopy is that with the first two methods it is not necessary for the specimens to be completely flat. The ATR-IR needs perfectly flat enamel specimens which means that the aprismatic enamel is removed and this might result in slightly different reactions to the bleaching agents.

Previous studies of Raman spectra in the quantification of molecules in enamel have utilized the intensity of the maximum peak, given in arbitrary units (9,12,17). The present study calculated the area under the curve in order to perform quantitative analysis, since according to Gilchrist *et al.* (7), this is the most precise method to compare data because it isolates the signal produced by the studied molecules and excludes the spectrum produced by the incident light and darkness coming from the CCD detector. Signal intensity of the carbonate groups is directly proportional to its concentration in dental enamel, thus it can be used to measure the effect of dental bleaching on the composition of this structure (11).

An important limitation of our study is that specimens were stored in distilled water instead of artificial saliva. Saliva contains phosphate, which has the potential of reverting the effect caused by the bleaching process and acting as an alkalizing agent. Despite of that, we decided against the use of artificial saliva since its exact composition is rarely reported by the manufacturer and it could introduce a new uncontrolled variable in the study.

Another important limitation in this investigation is that the specimens proceeded from different individuals, which means that the initial phosphate and carbonate values differed from each other. This might imply that some specimens might be more prone to losing minerals than others. However, all specimens were distributed in a randomized manner, so this limitation was compensated.

Further investigations might include analysis of in-office bleaching and their impact on phosphate, an also the influence of bleaching agents on other trace materials.

## Conclusions

Within the limitations of this study it can be concluded that home bleaching agents caused a carbonate loss, whilst the in-office bleaching agents increased the molecule concentration.
